# Accelerating segmentation of fossil CT scans through Deep Learning

**DOI:** 10.1038/s41598-024-71245-1

**Published:** 2024-09-09

**Authors:** Espen M. Knutsen, Dmitry A. Konovalov

**Affiliations:** 1 College of Science and Engineering, James Cook University, Townsville, QLD 4811 Australia; 2 Queensland Museum Tropics, Townsville, QLD 4810 Australia

**Keywords:** Palaeontology, Information technology, Scientific data

## Abstract

Recent developments in Deep Learning have opened the possibility for automated segmentation of large and highly detailed CT scan datasets of fossil material. However, previous methodologies have required large amounts of training data to reliably extract complex skeletal structures. Here we present a method for automated Deep Learning segmentation to obtain high-fidelity 3D models of fossils digitally extracted from the surrounding rock, training the model with less than 1%-2% of the total CT dataset. This workflow has the capacity to revolutionise the use of Deep Learning to significantly reduce the processing time of such data and boost the availability of segmented CT-scanned fossil material for future research outputs. Our final Unet segmentation model achieved a validation Dice similarity of 0.96.

## Introduction

In the past few decades, Computed Tomography (CT) scanning of fossil material has become the tool of choice for most palaeontologists wanting a non-destructive way to extract fragile fossils from their encasing matrix or to investigate their internal anatomy^1–4^.

With the introduction of conventional, synchrotron, and neutron micro-CT, datasets have become increasingly larger and more detailed, resulting in substantially longer post-processing times, in particular with respect to data segmentation^2,4–6^ With palaeontological material, where the density differences, and consequently X-ray image contrast, between the surrounding rock matrix and the fossil itself are usually very low, manual image segmentation is often the only way to digitally extract the Regions of Interest (ROIs) from the CT slice stacks^7,8^. This process can take weeks to months to complete, thus being a considerable bottleneck in making data available for research.

With the introduction of Deep Learning^9^, palaeontologists have started experimenting with methodologies for automatic image segmentation as a way to cut back on processing times for these large datasets. Recent studies on fossil invertebrate and vertebrate material suggest that much time can be saved using these new technologies^7,10^, but indicate that they currently require a significant amount of training input (manually segmented entire CT datasets) to accurately predict ROIs in more complex material, such as dinosaur skulls^7^.

Early Triassic vertebrate fossils from central Queensland (Australia) represent some of the southern hemisphere’s richest terrestrial and freshwater faunas from this time period^11^. Dating to shortly after the End-Permian Mass Extinction (EPME)^12,13^, material from these sites is integral to the understanding of ecosystem recovery in eastern Gondwana following this event. Many of the fossils are delicate, undistorted, three-dimensional remains of relatively small amphibians and reptiles (skulls < 50 mm in length), preserved in iron-enriched mudstone and fine sandstone^14,15^. Due to their size, fragility, and density of the rock, the, currently, best method for revealing their anatomy is through synchrotron X-ray micro-CT scanning. Over the last few years, close to 50 such scans have been done at the Imaging and Medical Beam Line (IMBL) at the Australian Synchrotron, and upwards of 20 new specimens are collected from localities across central Queensland every year.

**Figure 1:**
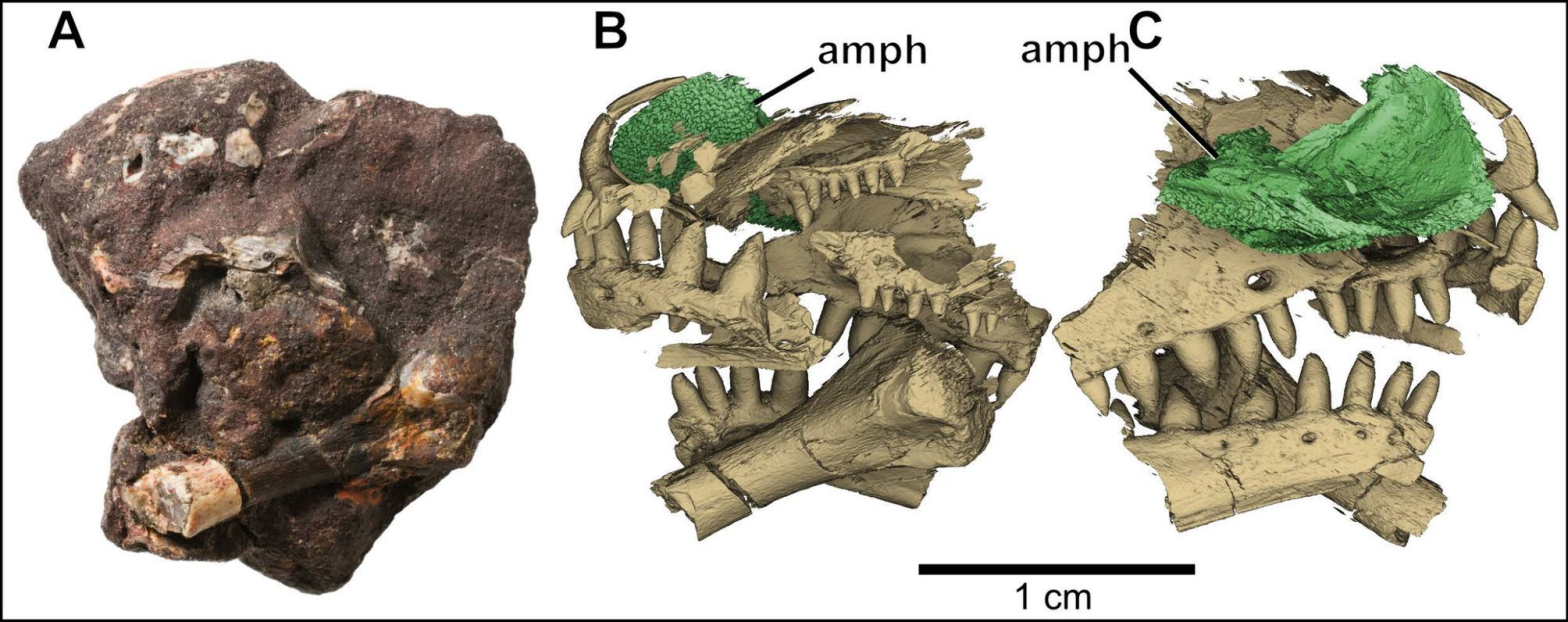
Photo of QMF60282 showing a partial limb bone and various cranial fragments at the surface (**A**), and 3D visualised results of final automated segmentation showing a partial procolophonid parareptile skull in left (**B**) and right (**C**) lateral views, and associated limb bone preserved inside. Note the presence of an ornamented amphibian cranial fragment (amph) in the anteriodorsal area, obscuring the right narial opening of the procolophonid.

Here we use synchrotron X-ray micro-CT scan data of a small specimen (Fig. 1A) from the Early Triassic Arcadia Formation of the Rewan Group in the Bowen Basin of central Queensland to test whether we can achieve highly accurate automatic segmentations from the entire dataset, using a very small amount of input slices, to significantly reduce the time required for manual segmentation of complex fossil vertebrate material.

**Figure 2:**
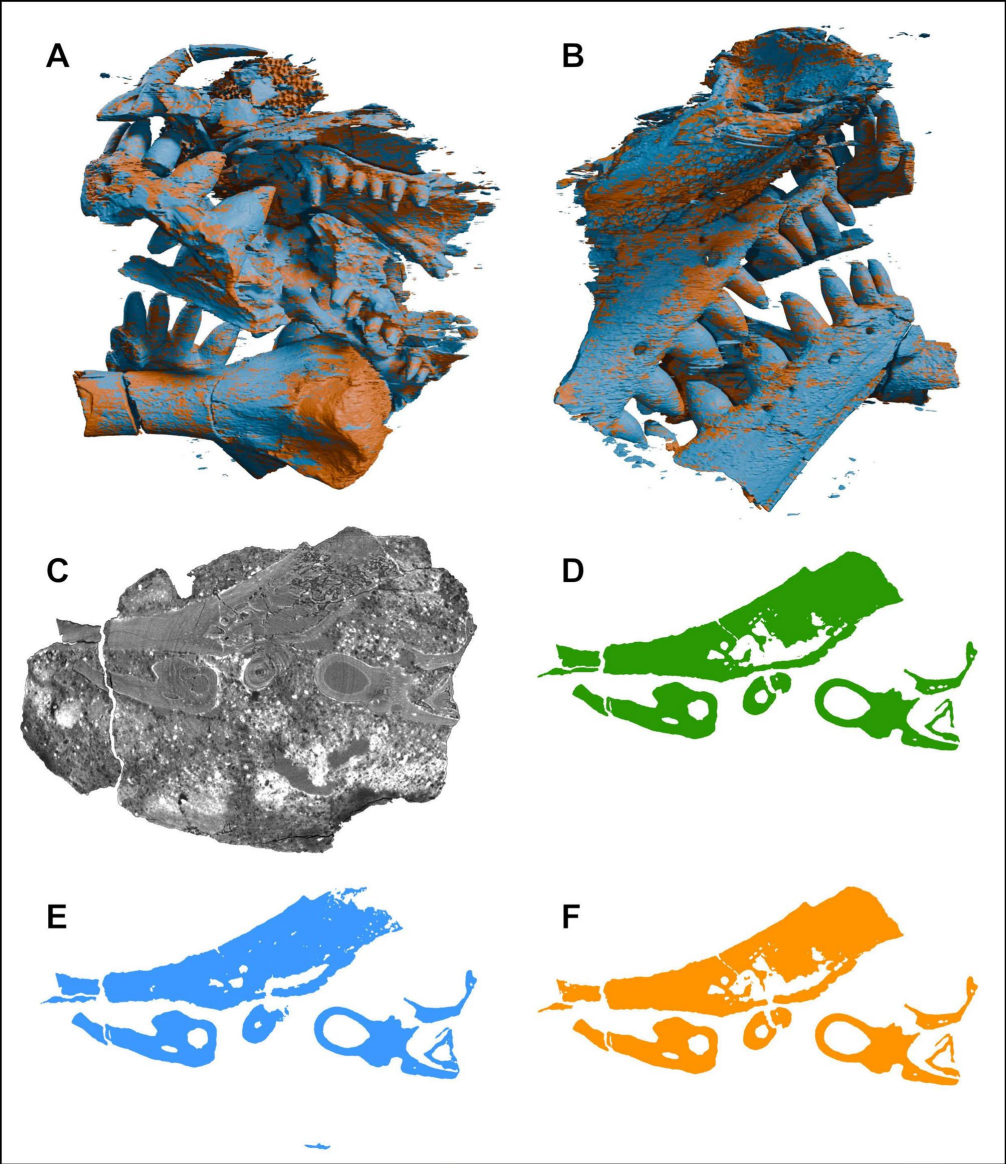
Superimposed 3D visualisation of the predicted ROIs from the first iteration in blue and the second iteration in orange (**A**,**B**), and X-ray CT scan slice number 750 (**C**) and its corresponding manual (**D**) and model predicted ROIs—first iteration (**E**) and second iteration (**F**).

**Figure 3:**
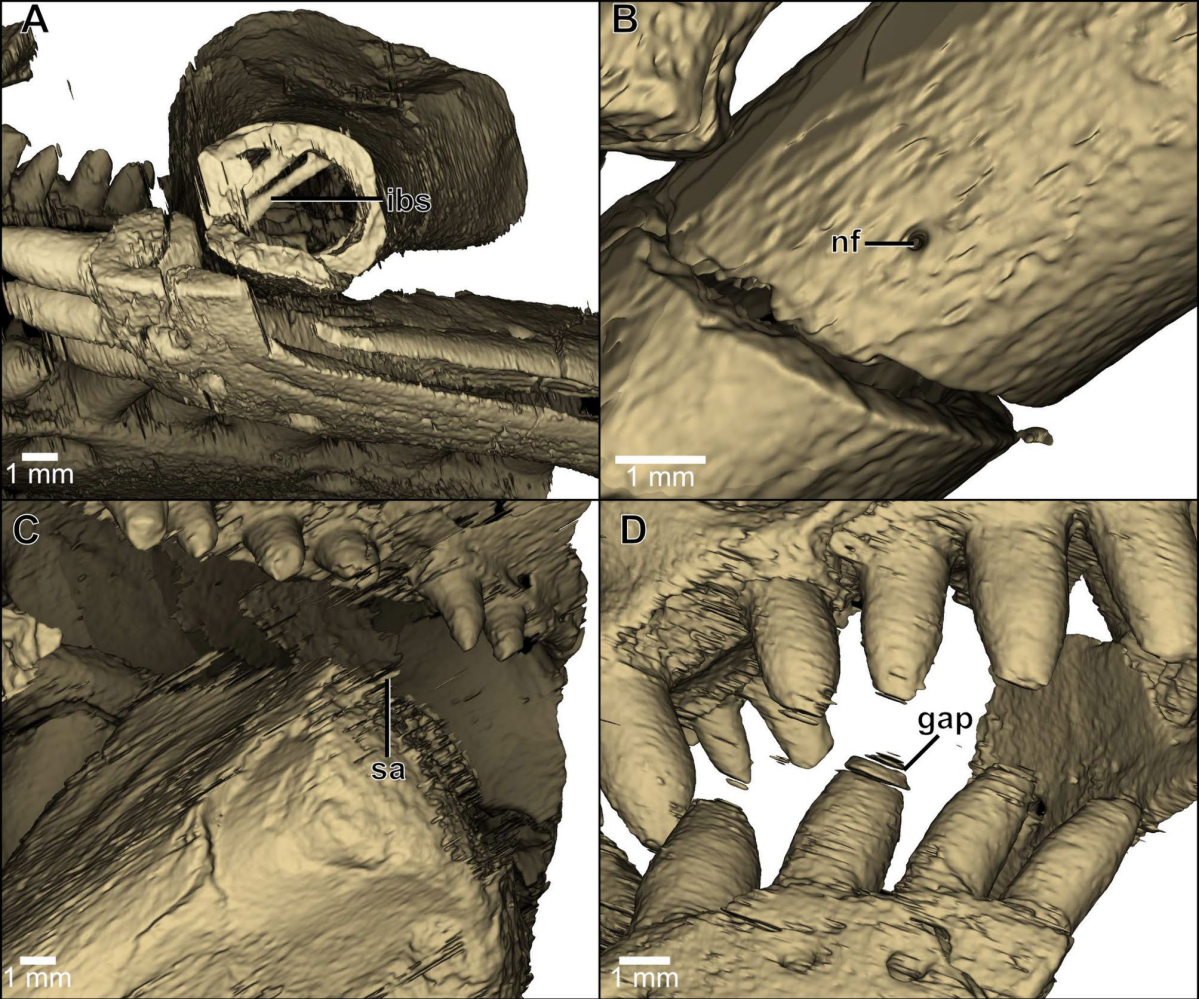
The high detail of the predicted ROI-stack makes visible minute and intricate details such as internal bone struts (**A**) and a $130\,\upmu \text {m}$ wide nutrient foramen in the limb bone (**B**). The effect of problem areas within the dataset are illustrated by minor streak artefacts (**C**) and gaps in the predicted ROIs (**D**). *ibs* internal bone strut, *nf* nutrient foramina, *sa* streak artefact.

**Figure 4:**
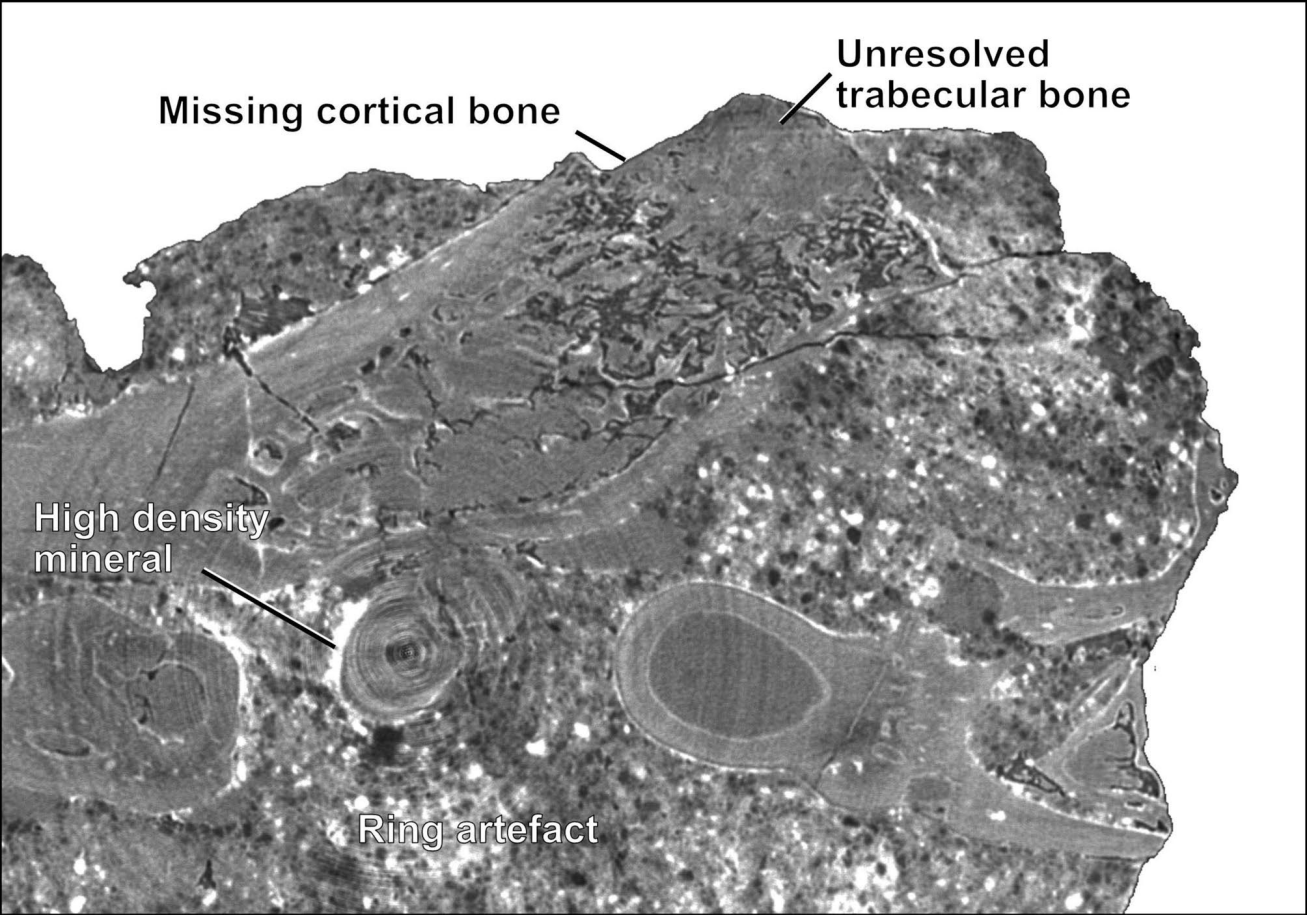
CT slice number 750 of QMF60282 illustrating some of the issues potentially affecting the accuracy of the model-predicted and/or manual segmentation.

## Results

Overall, the final model-predicted ROIs (visualised in Fig. 1B,C) result in a dataset which is very close to the accuracy obtained through manual segmentation (Fig. 2D,F). It reveals the intricate details of a small, partial procolophonid parareptile skull and associated limb bone (most likely *Eomurruna yurrgensis*^16^). Interestingly, a fragment of an amphibian cranium is preserved in the upper part of the specimen, obscuring the right naris of the procolophonid (Fig. 1A,B). This piece is thin and highly ornamented, which details have also been captured by the predicted ROIs.

The predicted stack of ROIs from the first iteration using nine input slices produced a highly accurate result for most of the different morphologies across the fossil, including teeth, jaws, palatal and exterior cranial bones (Fig. 2). However, the model deviates from the manual segmentation in areas of the interior of the limb bone and some of the finer details of the teeth and palatal bones (Fig. 2). There are also some false positives showing up as small fragments in different areas across the dataset (most easily observed in blue in the lower section of Fig. 2B).

We would like to highlight the significant productivity improvements observed even from the very first iteration, where the predicted ROIs required only relatively minor manual adjustments. This led to a substantial reduction in human effort compared to the extensive work involved in manually segmenting the ROIs from scratch.

Following the second training iteration, the accuracy of model-predicted ROIs are markedly improved in many of the regions where the initial model struggled, such as areas previously omitted and removing false positives (for comparisons see Fig. 2). Extremely fine details such as the internal bone struts and tiny nutrient foramina of the limb bone are resolved (Fig. 3A,B). However, some issues remain, causing minor streak artifacts and gaps in the dataset (Fig. 3B,C). Despite this, the final model produces a segmentation very close to that achieved through manual segmentation, and only requires minor manual adjustment for improved accuracy.

## Discussion

It is important to note that this study does not represent a start to finish workflow for capturing and interpreting CT datasets, but rather focuses on the segmentation stage of the process, which is currently the most significant bottleneck in making such data available for study. Therefore, a discussion of how to improve the quality of CT scans (which is a significant area of study in itself, including variables such as specimen pre-treatment, geometry, size, density, chemical composition, X-ray beam parameters, detector, exposure time, and image filtering) is beyond the scope of this paper.

Edie et al.^10^ showed that using the built-in deep learning “Segmentation Wizard” in Object Research Systems (ORS) Dragonfly Inc. (2022), they could achieve model’s accuracy score of up to 0.97 (Dice 0.13) using fewer than five training slides and 2.5D (3 slices) input dimensions (effectively 15 slices but still only 5 manually segmented ROIs) for fossil bivalve material. However, the morphology of bivalves is significantly less complex than that of a vertebrate skull. In contrast, Yu et al.^7^ used 7986 training slices to achieve mean Dice values of up to 0.894 for three fossil embryonic protoceratopsian skulls.

In this study, the predicted ROIs by the model trained with the first 9 manually segmented CT slices exhibited very high accuracy (Dice 0.93) compared to manually segmented ROIs (Fig. 2). Where the model primarily appears to struggle with delineating the presence or absence of fossil material is in areas where very small/thin bones rapidly change shape across very few slides, such as the bones in the palate or the interior of the limb bone (Fig. 2C–E), or where matrix-to-fossil contrast is low. This includes a number of unidentified, very small bones (some only a few CT slices thick) which are distributed across different areas within the rock matrix (some seen in blue in the lower section of Fig. 2B). These issues might be solved by omitting from the training data bones too small relative to the CT scan resolution, or a higher CT scan resolution is needed to resolve these features appropriately.

Due to the high accuracy of the initial Deep Learning model, additional input slices could easily be produced by manually fine-tuning the predicted ROIs. When adding an additional 9 manually segmented CT slices to the input dataset (a total of 18 slices out of 2159), there is a marked improvement in Dice (from about 0.93 to 0.96) and reduction of streak artefacts and gaps (Figs. 2A,B and 3C,D). However, minor errors persist in some areas, perhaps suggesting ambiguity in the manually segmented training data and/or possibly due to one or more of the following issues being present in the CT scan: (1) areas displaying scanning artefacts, e.g. beam hardening or ring artefact (both considered low in this example); (2) the presence of high-density materials (e.g. iron) causing image over-exposure; (3) very low fossil-to-matrix contrast and/or scanning resolution insufficient to objectively separate fossil from rock; (4) where cortical (surface) bone has eroded away, exposing less visually obvious cancellous/trabecular (spongy) bone; and (5) where bone complexity changes rapidly over a few slices or the presence of very small bones only a few slices in thickness (Fig. 4); where points 3–5 appear to have the most impact on the results in this example. These are issues also faced during manual segmentation, and as such, not necessarily a result of limitations in the model used here.

Nonetheless, the predicted result is an excellent and highly detailed foundation, requiring only very minor manual correction, thus saving (in this case) months of work. As such, this study provides a workflow for rapid segmentation of complex vertebrate fossil material, which will be applied to the high number of synchrotron micro-CT scans already available and continuing to be collected for the fossils being recovered from the Early Triassic rocks of Queensland, reducing the total required manual workload from years to weeks. It is also anticipated that this framework will be transferable to fossil material from other localities across the globe, with the potential to significantly bolstering the future availability of segmented CT-scanned fossils for continued research. However, due to the highly technical nature of the presented methodology, we recommend collaboration between the data user and a Deep Learning specialist for application on other datasets.

## Materials and methods

QMF60282 (Queensland Museum Collection) consists of a single cemented fine sand to mud rock fragment measuring approximately $20 \times 20 \times 10$ mm, collected from the Early Triassic Arcadia Formation in central Queensland, Australia. A partial limb bone and minor cranial fragments are the only fossil material visible at the surface. No preparation or pretreatment of the specimen was performed prior to the collection of this dataset. The specimen was CT scanned at the IMBL at the Australian Synchrotron in 2020, at 51 keV with a monochromatic beam, producing a stack of 2159 image slices measuring $2560 \times 2560$ pixels, and a voxel size of $10\,\upmu \text {m}$. Post-processing of the CT raw data, including ring artefact filtering, was done using software developed at and internal to IMBL at the Australian Synchrotron. Although the authors recognize the availability of further filtering for the removal of ring artefact (e.g. Wang et al.^17^) this was not considered necessary in this example, as the interference by the remaining ring artefact is relatively low over the areas of interest.

Across the visible extent of QMF60828 within the CT image stack, every 200th slice was manually segmented for Regions of Interest (ROIs) using Affinity Photo 2.4.1. The presence of air and rock matrix was scored as 0 (black), while fossil material was scored as 1 (white).

The initial training data consisted of 9 manually segmented slices (8 for training and 1 for validation). Following the first model training and automated ROI prediction, the model-generated ROI stack was used as a starting point for manually producing a further 9 input slices from mid-way between each slice in the original training data, effectively producing an overall training input from every 100th slice giving 18 slices in total.

The model-predicted ROI image stacks were visualised in 3D Slicer 5.7.^18^ and additional images of exported 3D meshes for model iteration comparisons were produced in Blender 4.0.2.

### Deep learning model

A deliberate effort was made to refrain from making unnecessary changes to the conventional deep learning models and techniques. This approach was driven by the desire to ensure that the training and prediction pipelines presented could be easily replicated. Below are the key features of our pipelines:

A UNet^19^ segmentation model was chosen as the foundational architecture due to its compatibility with various image feature encoders, facilitated through a widely-used GitHub repository of segmentation models^20^. A particularly useful aspect of the Yakubovskii’s library^20^ was its capacity to integrate with the extensive array of deep learning image classifiers found in the Hugging Face’s timm library^21^.

An exhaustive search for the optimal timm^21^ image encoder was beyond the scope of this project. Therefore, we relied on an educated guess informed by one author’s (D.A.K.) extensive experience in international Deep Learning and Machine Learning competitions^22^. We focused on exploring a series of EfficientNet-V2^23^ models, particularly those trained to recognize 21,000 different object classes in images.

To evaluate the performance of our segmentation models, we used the Dice^24^ coefficient, also known as the Sørensen-Dice coefficient or Dice similarity coefficient. This statistical metric measures the similarity between two sets, scoring a perfect one for exact overlap and zero for no overlap at all.

During the model training, we monitored the *validation* Dice metric on predictions for a single CT slice that was excluded from the training dataset, which consisted of either 8 or 17 slices. We employed progressively larger models from the EfficientNet-V2^23^ series as image encoders within our UNet segmentation model. Ultimately, the largest model, identified through its timm^21^ library code as tf_efficientnetv2_xl_in21k, proved to be the most accurate when measured by the validation Dice. Then, we incrementally increased the number of channels in the UNet decoder based on the improvements in the validation Dice metrics, culminating in a final configuration of [512, 256, 128, 64, 32] for the UNet decoding channels.

For the initial round of training, two extreme cases were evaluated. The first involved using the simplest region of interest (ROI) mask as the validation slice, while the second involved holding out the most complex ROI shape for validation. Given the limited dataset of only 8 training ROIs, the inclusion or exclusion of the most complex ROI significantly influenced overall prediction quality, depending on whether it was used for validation or included in the training subset. Consequently, both the first and second-stage models were trained on all available ROIs, excluding the simplest ROI, which was reserved for validation purposes. Therefore the second-stage validation Dice of 0.96 was likely to be an upper limit rather than an expected average Dice for the remaining slices without the manually created ROIs.

The CT slices featured 2560 rows and 2560 columns of pixels. Due to their large size combined with the large final model, these slices could not be used for training on the Nvidia RTX 3090 GPU available for this project. Consequently, for the final Unet models and throughout the training process, we utilized a training crop size of $512 \times 512$, loading 8 crops per batch. However, for prediction or inference, the full $2560 \times 2560$ CT slices were used. During training, the random cropping of $512 \times 512$ sections was strategically performed to ensure that each crop included (if available in that slice) at least some positive fossil segmentation mask pixels, thereby maintaining the relevance and balance of the training data.

The focus of this study was to optimize the human annotation effort and time. Segmenting the fossil-containing slices manually emerged as the primary time-consuming task, often requiring up to an hour per slice. In contrast, visually scanning and documenting the ranges of slices without fossils proved to be a much simpler task, typically taking just a few minutes. Consequently, this study leveraged a substantial number of negative samples with known zero fossil masks. To maintain a balance between the frequencies of positive and negative samples presented in training, we introduced a ratio of 2 random negative crops for every set of 8 (or 17) fossil-containing crops in each epoch—an epoch being a complete pass through all the available samples.

All models were trained using the following parameters: 5,000 epochs, a weight decay of 0.01, and the AdamW^25^ optimizer with a cosine annealing learning rate schedule. The initial learning rate was set at 0.0003125, calculated using AdamW’s heuristic $initialLearningRate = 0.01 * BatchSize / 256$, where $BatchSize=8$. Learning rates for the encoder layers were reduced by a factor of 10. The primary loss function used was Binary Cross Entropy. We used a learning rate warmup over the first 10 epochs, progressively increasing the learning rate from zero to the initial learning rate. The validation Dice metric was continuously monitored, and the model achieving the best validation Dice score was saved to disk. Additionally, training incorporated Automatic Mixed Precision (AMP) to optimize performance. It took approximately 10 hours to train the final second-iteration model.

Given the limited number of fossil-containing training slices (either 8 or 17) and the use of a high-capacity Efficient Net encoder, extensive image augmentation became necessary. We employed the Albumentations^26^ library to apply a wide range of image distortions. For instance, each training image crop was subjected to random rotations (0–360 degrees) and random flips. Without these augmentations, the model quickly overfitted the training slices, leading to high training Dice scores that did not translate into improvements in validation Dice scores.

In medical segmentation models, it is common to utilize not only a single grayscale CT slice as a one-channel image but also to include adjacent slices on both sides, creating what is known as 2.5D models^27^. Consequently, we experimented with both 1-channel and 3-channel input images. Interestingly, our high-capacity model typically yielded slightly better validation Dice scores for the 1-channel images compared to the 3-channel images that incorporated the original slice and its two neighbouring slices.

During inference, we employed the test-time augmentation (TTA) technique, which involved generating predictions for all eight unique variations created by 90-degree rotations and flips, reflecting Dihedral Symmetry in mathematics. The predictions from these eight TTA variations were then averaged for each slice. With approximately 2000 original CT slices, executing TTA inference required about three hours on the project’s GPU.

## Data Availability

The presented final models, training and prediction source code in Python and PyTorch could be made available on request on commercial or academic terms. The CT datasets presented in this study can be found in online repositories at 10.5061/dryad.mw6m9064n. Please note that the datasets included therein are sufficient be used for taxonomical, morphological and taphonomical studies, and is part of ongoing active research. We therefore request that you please ask for consent from either the correspondence author Espen M. Knutsen (espen.knutsen@qm.qld.gov.au) or the Queensland Museum Geoscience collection staff prior to using this data for such work.
